# Implementation of Cognitive-Behavioral Substance Abuse Treatment in Sub-Saharan Africa: Treatment Engagement and Abstinence at Treatment Exit

**DOI:** 10.1371/journal.pone.0147900

**Published:** 2016-01-27

**Authors:** Hetta Gouse, Jessica F. Magidson, Warren Burnhams, Jocelyn E. Remmert, Bronwyn Myers, John A. Joska, Adam W. Carrico

**Affiliations:** 1 Department of Psychiatry and Mental Health, HIV Mental Health Research Unit, Cape Town, South Africa; 2 Department of Psychiatry, Massachusetts General Hospital (MGH), Boston, Massachusetts, United States of America; 3 Harvard Medical School, Boston, Massachusetts, United States of America; 4 City of Cape Town Health. Substance Abuse, Cape Town, South Africa; 5 South African Medical Research Council, Alcohol Tobacco and Other Drug Research Unit, Cape Town, South Africa; 6 School of Nursing, University of California San Francisco, San Francisco, California, United States of America; Tulane University School of Public Health, UNITED STATES

## Abstract

**Aims:**

This study documented the treatment cascade for engagement in care and abstinence at treatment exit as well as examined correlates of these outcomes for the first certified Matrix Model^®^ substance abuse treatment site in Sub-Saharan Africa.

**Design:**

This retrospective chart review conducted at a resource-limited community clinic in Cape Town, South Africa, assessed treatment readiness and substance use severity at treatment entry as correlates of the number of sessions attended and biologically confirmed abstinence at treatment exit among 986 clients who initiated treatment from 2009–2014. Sociodemographic and clinical correlates of treatment outcomes were examined using logistic regression, modeling treatment completion and abstinence at treatment exit separately.

**Results:**

Of the 2,233 clients who completed screening, approximately 44% (n = 986) initiated treatment. Among those who initiated treatment, 45% completed at least four group sessions, 30% completed early recovery skills training (i.e., at least eight group sessions), and 13% completed the full 16-week program. Approximately half (54%) of clients who provided a urine sample had negative urine toxicology results for any substance at treatment exit. Higher motivation at treatment entry was independently associated with greater odds of treatment completion and negative urine toxicology results at treatment exit.

**Conclusions:**

Findings provide initial support for the successful implementation the Matrix Model in a resource-limited setting. Motivational enhancement interventions could support treatment initiation, promote sustained engagement in treatment, and achieve better treatment outcomes.

## Introduction

Historically there has been a gap between research and implementation of substance abuse treatment in developed countries [1, 2]. This gap is further heightened in low-and-middle-income countries (LMICs) such as South Africa (SA), where there is limited access to empirically-supported treatments and a shortage of trained health workers to deliver evidence-based interventions [3, 4]. In such settings where resources are scarce and the need for treatment is large, it is critical to understand the implementation of evidence-based treatments to ensure clients are receiving effective and acceptable care [5].

SA’s Western Cape province has pushed towards implementing evidence-based approaches to substance abuse treatment because of the well-characterized methamphetamine or “tik” epidemic in this region. Since 2002, Cape Town has experienced a 150-fold increase in methamphetamine users presenting for substance abuse treatment [6]. Because this region is thought to be among those with the highest prevalence of methamphetamine addiction globally [6], research is needed to document the implementation of evidence-based substance abuse treatment.

In 2007, in response to the drastic increase in methamphetamine use in this region, the City of Cape Town developed a multi-sectorial alcohol and drug strategy to manage the rapidly growing drug and alcohol-related challenges. One of the objectives of this strategy was to improve access to evidence-based treatment services through providing the Matrix Model of outpatient treatment for stimulant use disorders [7] within primary health care in local peri-urban communities. Developed and extensively tested in the United States (US), the Matrix model is an evidence-based, 16-week outpatient cognitive-behavioral treatment that includes individual, group, and family sessions [8–10].

Few published reports have documented the implementation of the Matrix Model in LMICs. Thus, it is unclear whether evidence of the effectiveness of this model for US populations can be extended to countries such as SA, where structural factors (e.g., poverty, shortages of trained health care workers, inability to take extended leave from work, and long distances to care) are substantial barriers to engagement in substance abuse treatment [11]. The overarching goal of this study is to describe one of the first examples of Matrix Model implementation in one low-income, resource-limited community clinic in Cape Town. The primary objectives were to: 1) document the services provided and characteristics of the treatment-seeking population; 2) describe the “cascade” of treatment engagement from screening through completion; 3) examine abstinence at treatment exit using urinalysis results; and 4) explore sociodemographic and clinical correlates of treatment engagement and abstinence at treatment exit.

## Method

### 2.1. Setting

This study took place at the first certified Matrix Model site in Africa. The site is based within a community health center in a peri-urban area outside Cape Town. The substance abuse treatment program was launched in 2008, and in 2010 it was certified as a Matrix Model^®^ ‘program of excellence’ [12]. The clinic serves a low-income, largely “coloured” (an apartheid classification for ‘mixed race’ that is still in use) community in the surrounding area; however, clients are not limited to this catchment area due to the lack of substance abuse treatment services in the region. Although the program primarily serves methamphetamine users, many clients use multiple substances including alcohol, heroin, methaqualone (mandrax), and cannabis.

### 2.2. Description of Matrix program

This outpatient treatment program is based on the standardized Matrix Model^®^ [7]. The core 16-week program consists of 8 sessions of early recovery (with two early recovery groups per week for the first four weeks), 32 sessions of relapse prevention (two relapse prevention groups per week for sixteen weeks), and other optional weekly individual or conjoint sessions. Clients typically attend two to four sessions per week. At least one mandatory random urine drug panel test screen is required on a weekly basis from all clients; for clients who are primary alcohol users, an alcohol breathalyzer test is required.

The first point of contact is by a non-appointment screening visit conducted by a lay counselor. Starting in November 2011, the World Health Organization Alcohol, Smoking, and Substance Involvement Screening Test (ASSIST) [13], the Stages of Change Readiness and Treatment Eagerness Scale (SOCRATES) [14] and a urine drug test with alcohol breathalyzer were administered at the screening visit. Referrals to other services are made for clients experiencing severe withdrawals from alcohol, heroin users with difficulties remaining abstinent on an outpatient basis (referred for detoxification services and residential treatment), those experiencing psychosis, where there is serious suicide risk (referred to a psychiatric hospital), or those unable to attend an intensive outpatient program (e.g., for work reasons). If the client is suitable for the Matrix Model program, the first session (third contact) is generally attended within two days of enrollment.

#### 2.2.1. Matrix site therapists

The lay counselor is trained in basic substance abuse and mental health counseling skills. The minimum educational requirement for a therapist is a four-year degree in social work or counseling psychology; and for the Key Supervisor, a Masters level degree in a related field. Therapists receive Matrix Core Training from US-based Matrix program trainers and complete a 5-day Motivational Interviewing Network of Trainers (MINT)-certified Motivational Interviewing course. The program Key Supervisor, experienced in implementation of the Matrix Model, provides individual case supervision and Matrix supervision sessions once per month.

### 2.3. Procedures

#### 2.3.1. Data extraction

We retrospectively evaluated services provided at this clinic from one-year post inception (June 2009) until May 2014. The cut off point for inclusion in the evaluation was 4 months prior to the start of data extraction so that all included participants had the opportunity to complete the 16-week program. Informed consent was waived on the basis of it being a retrospective chart review and de-identification of data. Data used in this research project were extracted from chart reviews conducted on all existing intake and discharge assessments collected during routine care. All data identifying clients were removed from clinical records before research activities were implemented. The chart-review was approved by the University of Cape Town Human Research Ethics Committee and City of Cape Town Health Department.

#### 2.3.2. Measures

*Sociodemographics and treatment history*: Age, ethnicity, gender, education level, relationship status, and employment, and number of previous treatment episodes were assessed.

*ASSIST V.3.0 [13]:* The ASSIST assesses level of substance use severity for alcohol, tobacco and illicit drugs. A total score is calculated for each substance that indicates level of substance use involvement, which is then used to categorize the person into low (0–3), medium (4–26) and high risk (>27) of substance-related health problems for illicit drugs, and low (0–10), medium (11–26) and high risk (>27) for alcohol.

*SOCRATES [14]*: The SOCRATES assesses readiness for change among alcohol and substance users, and has been found to have good internal consistency and reliability across diverse samples. It yields three composite scores ranging from 10 (very low) to 90 (very high): *Recognition* indexes acknowledgment of substance use related problems (α = .85–95). *Ambivalence* measures degree of uncertainty about changing substance use (α = .60–.88). *Taking Steps* provides information regarding the degree to which individuals are taking concrete actions towards changing substance use (α = .83–.96) [14].

*Clinical outcome variables*: *Treatment initiation and engagement*. Treatment initiation was defined as attending at least one group or individual session. To define *treatment engagement*, clinically meaningful cut-offs were based on the existing evidence-based Matrix Model program structure in accordance with the City’s Matrix Key Supervisor (WB): 1) attending at least four group sessions (2 weeks), 2) attending at least eight group sessions (i.e., *completing early recovery*; one month); 3) attending at least sixteen group sessions (2 months); 4) attending at least 24 groups sessions (three months); and 5) completion of the full 16-week program (i.e., *completing relapse prevention*; 4 months).

*Urine tox screen results at treatment exit*. Urine drug tests were administered on a weekly basis for all clients using a Drugs of Abuse Panel Test Card for amphetamine, benzodiazipine, cocaine, opiate, marijuana (THC). Tox screen results at treatment exit were assessed using urine drug test results (positive or negative) for all substances in the last two weeks of each client’s clinic attendance; any use in the last two weeks was defined as a positive screen, and no use was defined as a negative screen.

#### 2.3.3. Statistical analysis

To describe the cascade of substance abuse treatment engagement, the number of group sessions attended was calculated to document the markers of treatment engagement described above. Next, frequencies of urine tox screen results were calculated during the two weeks before each client exited treatment. Finally, sociodemographic and clinical correlates of treatment outcomes were examined using logistic regression, modeling treatment completion and abstinence at treatment exit separately.

## Results

### 3.1. Descriptive characteristics of sample

From June 2009 through May 2014 a total of 2,233 clients had a screening visit at the Matrix Model^®^ program. Clients were 66% male (*n* = 1,471), 97% “coloured” (*n* = 2,161), 86% unemployed (*n* = 1,912), 68% single (*n* = 1,507), and mean age of 28.3 (*SD =* 7.6) years. Methamphetamine (61%; *n* = 1,329) and heroin (24%; *n* = 534) were the two most commonly reported primary substances of abuse. For 76% (*n* = 1,704) of the sample, this was the first reported substance abuse treatment episode. Of those who received the ASSIST (*n* = 1,041), 76% were in the moderate- to high-risk categories for amphetamines (*n* = 790) and 28% in the moderate- to high-risk categories for opioids (*n* = 288). More than half (58%) of clients were in the moderate to high-risk categories for more than one substance.

### 3.2. Cascade of treatment engagement

**Fig 1:**
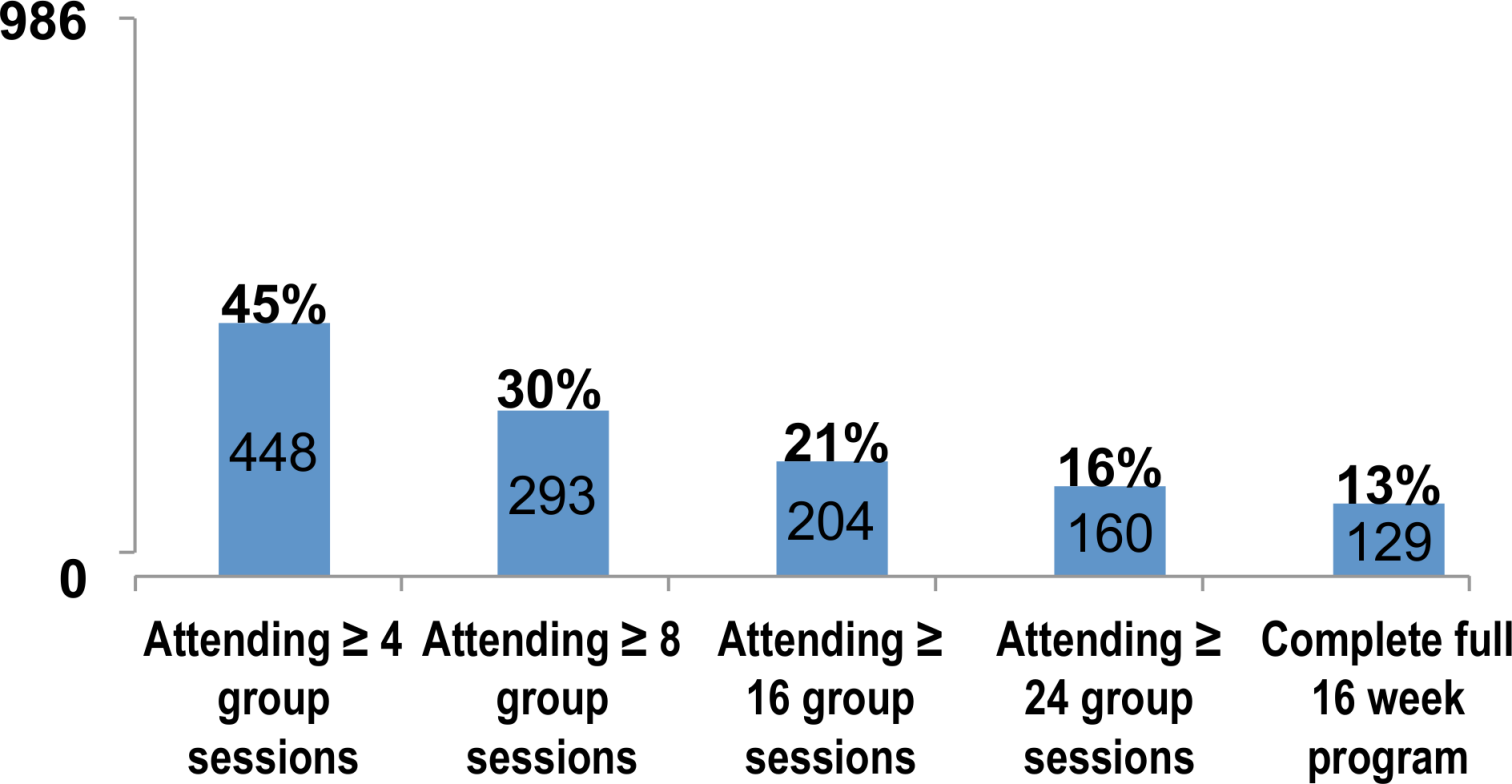
Cascade of engagement in care (N = 986). Attending ≥ 8 groups sessions = completion of early recovery; Complete full 16 week program = completion of relapse prevention.

Fig 1 illustrates engagement in outpatient Matrix Model^®^ substance abuse treatment among clients who initiated treatment. Of the 2,233 clients who completed screening, 44% (*n* = 986) initiated treatment. Among those who initiated treatment, less than half (45%; *n* = 448) completed two weeks of the program, approximately one-third (*n* = 293) completed 1 month of treatment, which marks completion of the early recovery module, and one out of eight (13%; *n* = 129) completed the full program, which marks the completion of relapse prevention.

### 3.3. Urine toxicology screening results

Of the 986 clients who initiated treatment, 564 (57%) had a urine tox screen result at treatment exit, which could have occurred at any time during the 16-week program. Of those, 304 (54%) had a negative result at treatment exit for all substances. Of those who completed at least eight group sessions (first month of treatment) and had urine tox screen results available in their last two weeks of treatment (*n* = 260), 69% had a negative result at treatment exit for all substances (*n* = 180).

### 3.4. Multivariable models

As shown in Table 1, only treatment motivation as measured by the SOCRATES was significantly associated with treatment completion. Higher scores on the “Taking Steps” and “Recognition” subscales as well as lower scores on the “Ambivalence” subscale were independently associated with greater odds of treatment completion. As shown in Table 2 higher scores on the “Taking Steps” subscale were independently associated with greater odds of abstinence at treatment exit, and greater severity of methamphetamine use was independently associated with lower odds of abstinence at treatment exit.

**Table 1:** Correlates of treatment completion (N = 129).

	B	S.E.	Wald	df	Sig.	Exp	95% C.I.		
							Lower	Upper
Sex	.27	.43	.40	1	.52	1.31	.56	3.06
Employment	.80	.75	1.12	1	.29	2.23	.50	9.8
Age	.03	.02	1.86	1	.17	1.03	.98	1.08
**Socrates**								
Recognition	.12	.06	3.96	1	.04	1.13	1.0	1.28
Ambivalence	-.14	.06	4.74	1	.02	.86	.76	.98
Taking Steps	.12	.04	7.00	1	.00	1.12	1.03	1.23
Previous Treatment Episode	-.08	.44	.04	1	.84	.91	.38	2.18
**ASSIST**								
Methamphetamine	-.00	.01	.00	1	.95	.99	.96	1.0
Marijuana	-.01	.01	.64	1	.42	.98	.95	1.02
Alcohol	-.01	.02	.18	1	.66	.98	.93	1.04
Opioids	-.03	.02	3.01	1	.08	.96	.92	1.00
Constant	-5.36	1.18	20.53	1	.00	.00		

Note: Dependent variable (treatment completion) was coded as 0 = less than 16 weeks

1 = greater than 16 weeks.

ASSIST and SOCRATES scores were mean centered for ease of interpretation.

**Table 2:** Correlates of a negative urine toxicology result in last two weeks of treatment (N = 304).

	B	S.E.	Wald	df	Sig.	Exp	95% C.I.	
							Lower	Upper
Sex	.37	.30	1.52	1	.21	1.45	.80	2.65
Employment	.29	.41	.48	1	.48	1.34	.58	3.04
Age	.01	.02	.48	1	.48	1.01	.97	1.05
**Socrates**								
Recognition	.03	.03	.87	1	.35	1.03	.96	1.10
Ambivalence	-.06	.05	1.49	1	.22	.93	.84	1.04
Taking Steps	.10	.02	12.63	1	.00	1.10	1.04	1.16
Previous Treatment Episode	-.44	.31	2.04	1	.15	.64	.34	1.17
**ASSIST**								
Methamphetamine	-.02	.01	5.24	1	.02	.97	.95	.99
Marijuana	-.01	.01	1.37	1	.24	.98	.96	1.00
Alcohol	.03	.01	3.83	1	.05	1.03	1.00	1.07
Opioids	-.00	.01	.21	1	.64	.99	.96	1.02
Constant	-.87	.76	1.32	1	.24	.41		

Note: Dependent variable (drug test result in last 2 weeks of treatment) was coded as 0 = positive, 1 = negative.

ASSIST and SOCRATES scores were mean centered for ease of interpretation.

## Discussion

Findings provide one of the first opportunities to describe implementation of the evidence-based Matrix Model cognitive-behavioral substance abuse treatment program in a resource-limited setting in SA. To our knowledge, there have been limited examples, if any, of studies that have documented the implementation of evidence-based substance abuse treatment in a resource-limited setting in Sub-Saharan Africa, where many structural barriers to treatment exist and there are limited options for evidence-based care [15, 16]. This Matrix site provides treatment in an area of SA with high rates of polysubstance use and one of the highest rates of methamphetamine use in the world [10].

Over a period of 72 months, the clinic had attrition rates comparable to that of published research conducted in industrialized nations. For example, the attrition rate at the Matrix Model clinic site between the initial request for service at screening and treatment initiation was 56%, which is consistent with rates between 29%–63% reported in the literature [2]. Similarly retention after 30 days of treatment was 30%, consistent with rates of 34% reported in the literature for patients with no prior treatment [2, 17]. Overall, findings highlight that enhancing initiation and engagement in treatment is an important area for further study to optimize the benefits of substance abuse treatments in LMICs and industrialized nations.

The opportunity for a drop-in screening visit at this site increases rates of initial contact with the site (versus starting with a visit that requires an appointment), which in turn may also inflate the rate of dropout seen here from screening to the first treatment session. That being said, the population this Matrix site serves (and other populations in resource limited settings) face substantial challenges around treatment initiation and retention including: gaining employment, the impact of gang related activities in the clinic neighborhood, lack of child-care facilities, migration, transport related issues [18], and few other evidence-based substance abuse treatment referral options [19]. The influence that these factors have on treatment retention and possible solutions to overcome these structural barriers should be addressed in future clinical research in LMICs and industrialized nations.

It should also be noted here that early termination of treatment does not equate to treatment failure. Clients may have terminated treatment for reasons other than relapse, including finding employment. Further, the rate of negative drug tox screens for all clients who were tested two weeks prior to treatment termination (54%) and those who completed at least one month of treatment (69%) suggest that even limited interaction with the Matrix Model program may assist clients with achieving some degree of abstinence. The association of degree of engagement with treatment outcomes should be examined in future research.

Motivation emerged as a key psychological factor that predicted greater engagement in treatment and better treatment outcomes. Those who reported awareness of substance-related problems (i.e., Recognition) and taking concrete steps to reduce substance use (i.e., Taking Steps) at treatment entry had greater odds of completing the full 16-week substance abuse treatment program. Greater uncertainty about setting goals for changing one’s relationship with substances (i.e., Ambivalence) was independently associated with decreased odds of treatment completion. Findings point to the need for expanded efforts to enhance motivation for treatment, including approaches like contingency management that provide motivational incentives as positive reinforcement for abstinence that could also mitigate the effect of structural barriers to remaining engaged in treatment in this resource-limited setting [20–23].

## Limitations

The study findings must be interpreted in the context of important limitations, including those inherent in this being a retrospective chart review. Urine drug screen data was available only for the last two weeks of program attendance, and as such we were unable to document the number of clients at each step in the cascade with negative urine drug test results. Additionally, we could not assess reasons for ‘drop-out’ and were limited to only examining the measures included in routine clinical assessments. Tobacco use is not treated explicitly in this clinic setting and was therefore not included in our analysis. Alcohol users had greater odds of abstinence at treatment exit, but this may be due to the fact that breathalyzer data were not extracted. Further research is needed to examine the extent to which those who do not have stimulant use disorders achieve comparable benefits from the Matrix Model in this setting.

## Conclusions

Findings provide initial support for the successful implementation the Matrix Model in a resource-limited setting. We found that clients with heterogeneous, severe substance use profiles were making contact with the program for their first treatment episode. Rates of initiation and engagement were comparable to the implementation of substance abuse treatment in industrialized nations. Motivational enhancement interventions could support treatment initiation, promote sustained engagement in treatment, and achieve better treatment outcomes. Future mixed methods research should examine whether the barriers faced to retention in care are distinct in this setting.

We hope this work spurs further efforts to address the many remaining empirical questions about how to optimally deliver evidence-based substance abuse treatment in resource-limited settings where huge clinical needs exist, particularly related to questions regarding how to enhance motivation and address structural barriers to remaining engaged in care.
